# The impact of Mood on Sports Flow State in football players: a chain mediating model of Psychological Resilience and Achievement Motivation in Competition

**DOI:** 10.3389/fpsyg.2025.1523400

**Published:** 2025-05-09

**Authors:** Jiang Li, Xiaofei Pan

**Affiliations:** Chengdu Sport University, Chengdu, China

**Keywords:** Mood, Sport Flow State, Psychological Resilience, Achievement Motivation in Competition, chain intermediary

## Abstract

**Objectives:**

To explore the relationship between Mood and Sport Flow State in football players, and to clarify the mediating roles of Psychological Resilience and Achievement Motivation in Competition.

**Methods:**

Convenience sampling was used to conduct a questionnaire survey on 388 football players. Use Profile of Mood State (POMS), Smooth Experience Scale-2 (SES-2), Connor Davidson Resilience Scale (CD-RISC), and Achievement Motivation Scale (AMS) to evaluate the Mood, Sport Flow State, Psychological Resilience, and Achievement Motivation in Competition of football players. Pearson correlation analysis and mediation analysis were used to investigate the relationship between the four variables of Mood, Sport Flow State, Psychological Resilience, and Achievement Motivation in Competition among football players.

**Results:**

Football players’ mood has a negative impact on Sport Flow State, Psychological Resilience, and Achievement Motivation In Competition, while Sport Flow State, Psychological Resilience, and Achievement Motivation In Competition have a mutually positive impact. Psychological Resilience has a positive impact on Achievement Motivation in Competition. The mediating effect of Psychological Resilience (POMS → CD-RISC → SES-2) accounts for 26.9% of the total effect (r = −0.014, 95% CI −0.020∼−0.009), Achievement Motivation in Competition (POMS → AMS → SES-2) accounts for 17.3% of the total effect (r = 0.009,95% CI −0.013∼−0.005), and the chain mediating effect of Psychological Resilience and Achievement Motivation in Competition (POMS → CD-RISC → AMS → SES-2) accounts for 32.6% of the total effect (r = −0.017, 95% CI −0.022∼−0.013).

**Conclusion:**

Football player Mood has a significant negative impact on Sport Flow State, but its actual predictive ability for Sport Flow State is very small. Psychological Resilience and Achievement Motivation in Competition play a chain mediating role in this relationship.

## 1 Introduction

Mood refers to a weak and persistent psychological state of an individual, reflecting their psychological state and consciousness, usually without clear, usually without a clear direction. At the same time, Mood has a diffuse characteristic, which not only reflects the psychological state of an individual at a specific stage, but also forms a certain Mood throughout the individual’s entire mental activity (Zhu, 1995). Related studies have shown that Mood status has a significant impact on athletes’ athletic performance, and positive Mood can improve the reaction speed and decision-making ability of football players, thereby contributing to improved athletic performance (Filaire et al., 2001). A negative Mood can easily lead to distraction, muscle tension, and uncoordinated movements, which in turn affect technical execution and motor performance (Chennaoui et al., 2016).

The Sport Flow State refers to the conscious state in which an athlete fully engages in a task and creates the optimal level of athletic performance (Jackson and Roberts, 1992). The concept of sports flow state comes from Csikszentmihalyi’s “Flow” concept based on the phenomenon of athletes’ psychological peak experience. Sports Flow State includes the following nine psychological characteristics: challenge skill balance, action consciousness fusion, clear goals, complete focus on the current task, sense of control, loss of self-awareness, time shift, clear feedback, and enjoyable experience (Csikszentmihalyi, 1990). Related studies have shown that there is a significant correlation between the Mood of football players and the occurrence of game fluency, especially for individuals with confidence and focused attention. Mood is more likely to help football players enter the Sport Flow State (Cai and Si, 2004). The Mood and Sport Flow State of football players are also influenced by multiple factors. Throughout the entire league cycle, factors such as home and away game experience, weather, audience and referees, and non-match factors can all affect the Mood of football players (Szczepaniak and Guszkowska, 2017). Related studies have also found that both team and individual sports have a relatively high level of flow when playing football (Strahler et al., 2010), indicating that football, as a high-intensity intermittent collective competitive sport, has significant individual or collective sports flow states. Meanwhile, a study suggests that Mood status has a significant impact on the athletic performance of soccer players. Maintaining a positive Mood among adolescent soccer players can improve their strength training efficiency and enhance their passing and receiving abilities during teaching competitions (Selmi et al., 2018). However, there is currently limited research on the correlation between the Mood of football players and their Sport Flow State, and further verification is needed to determine whether the Mood of football players affects their Sport Flow State. Therefore, the study proposes hypothesis 1: there is a significant negative correlation between the Mood and Sport Flow State of football players.

Psychological Resilience refers to an individual’s psychological trait of being difficult to defeat and their personal ability to produce advanced subjective achievements or objective manifestations when faced with challenging or threatening sports situations (Yang, 2014). Numerous studies have confirmed that athletes with strong Psychological Resilience can effectively regulate their psychological Mood before and after the competition, form a reasonable competitive mentality, and actively adjust their psychological state during the competition, exhibiting better competitive performance (Reinebo et al., 2024; Yang et al., 2020; Shang and Yang, 2021). Related studies have proposed that Psychological Resilience is a natural or acquired psychological advantage that has a significant impact on athletes’ performance in stressful situations. Psychological Resilience can interact to regulate the psychological state of low Moodal football players, forming proactive cognitive control and improving their sports psychological state (Wang and Jiang, 2018). Based on this, the study proposes hypothesis 2: Psychological Resilience mediates the relationship between Mood and Sport Flow State in football players.

Achievement Motivation in Competition refers to the psychological drive of athletes to achieve excellent results or success through competition, in order to gain self-worth and a sense of achievement. In the study of elite athletes, a strong Achievement Motivation in Competition is often regarded as an essential psychological quality for excellent athletes (Gould et al., 2002). Related studies have also shown that Achievement Motivation has a significant impact on the athletic performance of adolescent football players (Claudia and Conzelmann, 2014). Athletes with strong Achievement Motivation in Competition have stronger goal orientation and stronger psychological regulation drive under high-intensity situational conditions (Pol et al., 2012). Based on the theory of holistic development, relevant research conducted an Achievement Motivation survey on 97 top young football players and found that players with a high degree of intrinsic achievement orientation are more likely to exhibit high individual psychological stability (Zuber et al., 2015). Football players with strong Achievement Motivation have clearer sports goals and development plans, and perform better in sports (Garcia-Naveira et al., 2011). Based on this, the study proposes hypothesis 3: Achievement Motivation in Competition plays a mediating role in the Mood and Sport Flow State of football players.

**FIGURE 1:**
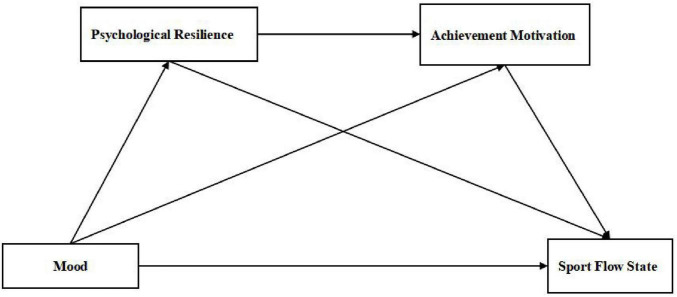
Chain mediation theory model.

In summary, based on the analysis of existing research, in order to more effectively explore the impact of Psychological Resilience and Achievement Motivation in Competition on the Sport Flow State of football players, a necklace style mediation model was constructed (Figure 1), proposing hypothesis 4: Psychological Resilience and Achievement Motivation in Competition play a chain mediation role in the impact of football players’ Mood on the Sport Flow State.

## 2 Research methods

### 2.1 Participants

The study used convenience sampling to distribute a survey questionnaire on football players to students majoring in sports training in sports colleges and universities. A total of 416 questionnaires were collected, and 28 invalid questionnaires were excluded. Finally, 388 questionnaires were selected for analysis, with an effective rate of 93.26%, including 296 male students (76.3%) and 92 female students (23.7%). The main reason for choosing students majoring in sports training is that this group is basically at the level of Chinese national second level football players or above, with a long football system training period and competition experience. More than 85% of the athletes have achieved top three results in youth football leagues at the provincial and above levels. The average age of the tested athletes was 18 ± 1.28, with the highest proportion of athletes receiving 6–9 years of systematic training, reaching 51.3%. Athletes with 10 years or more of training accounted for 16.5%, and athletes with 1–5 years of training accounted for 32.2%. In the composition of sports levels, first level athletes account for the largest proportion, reaching 74.3%, second level athletes account for 22.8%, and elite athletes account for 2.9%.

### 2.2 Research tools

#### 2.2.1 Profile of Mood State

The study used the Profile of Mood State (POMS) revised by Zhu Beili to measure the Mood state of participants within a week (Zhu, 1995). This scale includes seven components: anger, depression, energy, panic, fatigue, and self-esteem, using a Likert 5-point scoring system ranging from 0 to 4 points. Subtracting the sum of the scores of the five negative Mood scales from the sum of the scores of the two positive Mood scales, and adding a correction value of 100, the total score of negative Mood disorder is obtained. The higher the score, the stronger the negative Mood. The Cronbach’s alpha coefficient is an important indicator for measuring the internal consistency of a scale, and α = 0.80 indicates good internal consistency of the scale (Cronbach, 1951). The Cronbach’s alpha coefficient of this questionnaire in this study is 0.819.

#### 2.2.2 Smooth experience scale-2

Using Liu Weina’s revised Chinese version of Smooth Experience Scale-2 (SES-2) to measure the Sport Flow State of football players (Liu, 2009). This scale is divided into eight dimensions: enjoyable experience, clear goals, clear feedback, sense of control, loss of self-awareness, skill challenge balance, time transition, and action consciousness integration. Each dimension is answered using a 0–4 Likert 5-point scoring method, and the average score of the eight dimensions is taken as the final Sport Flow State score. The higher the score, the better the Sport Flow State. The Cronbach’s alpha coefficient of this questionnaire in this study is 0.826.

#### 2.2.3 Connor—Davidson Resilience Scale

The study used the Connor Davidson Resilience Scale (CD-RISC) revised by Yu and Zhang (2005) from The Chinese University of Hong Kong to measure the Psychological Resilience of football players. This scale consists of three factors: resilience, strength, and optimism, and is evaluated using a 0–4 point Likert 5-point scale. The total score of the scale is the sum of the scores of each item, and the higher the individual score, the higher their Psychological Resilience level. The Cronbach’s alpha coefficient of this questionnaire in this study is 0.841.

#### 2.2.4 Achievement Motivation scale

The study used the Achievement Motivation Scale (AMS) developed by Si et al. (2003) as a measurement tool for football players’ Achievement Motivation in Competition. This scale consists of two dimensions: social orientation and personal orientation, and is evaluated using a 1–6 point Likert 6-point scale. The total score of the scale is the average score of the social orientation dimension plus the average score of the personal orientation dimension, divided by two. The higher the score, the stronger the athlete’s motivation for social or personal achievement in sports. The Cronbach’s alpha coefficient of the questionnaire in this study was 0.837.

### 2.3 Statistical methods

Statistical analysis was conducted using SPSS 23.0 software, with measurement data expressed as mean ± standard deviation and count data expressed as number of people and percentage. Pearson correlation analysis was used to evaluate the correlation between the study variables, and Model 6 in the PROCESS macro program was used to perform chain mediation analysis on the relevant variables, with a test level of α of 0.05. Using the Beta coefficient to reflect the direction and strength of the relationship between variables, the larger the value, the stronger the mediating chain effect.

## 3 Results

### 3.1 Common method deviation test

This study used self-report method to collect data, which may have common methodological biases. Exploratory factor analysis was conducted using Harman single factor test (Zhou and Long, 2004), and the results showed that the first factor explained 29.36% of the variance. Therefore, the data in this study were not affected by significant common method bias.

### 3.2 Correlation analysis

The Pearson correlation analysis results of each variable (Table 1) showed that POMS score was negatively correlated with SES-2 score, CD-RISC score, and AMS score (r = −0.591, *P* < 0.01; r = −0.462, *P* < 0.01; r = −0.564, *P* < 0.01). This result validates Hypothesis 1: there is a significant negative correlation between Mood and Sport Flow State among football players, indicating that football players with negative Mood exhibit lower Sport Flow States. The SES-2 score was positively correlated with the CD-RISC score (r = 0.871, *P* < 0.01) and also positively correlated with the AMS score (r = 0.903, *P* < 0.01). The CD-RISC score is positively correlated with the AMS score (r = 0.902, *P* < 0.01). The higher the negative Mood level of football players, the lower their Sport Flow State. At the same time, strong negative Mood of football players may weaken individuals’ confidence in their own abilities, reduce Psychological Resilience when dealing with stress, and hinder the strengthening of Achievement Motivation in Competition. The above research results are consistent with previous research findings (Yang et al., 2020; Salmana et al., 2021; Nunu et al., 2023).

**TABLE 1:** Variable correlation.

Variable	POMS	SES-2	CD-RISC	AMS
POMS	1	–	–	–
SES-2	−0.591**	1	–	–
CD-RISC	−0.462**	0.871**	1	–
AMS	−0.564**	0.903**	0.902**	1

** Indicates *P* < 0.01.

### 3.3 Mediation effect test

**FIGURE 2:**
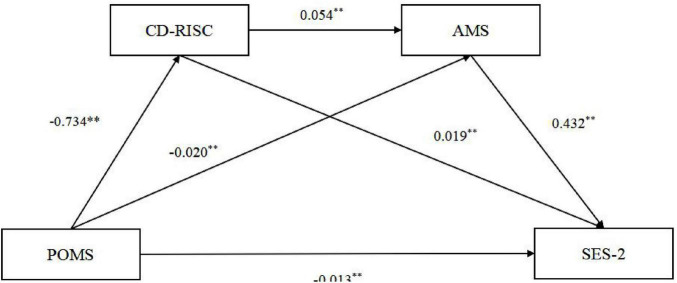
Chain mediation effect diagram.

The results of the mediation effect test (Table 2) show that the beta coefficient reflects the influence of one variable on another, while controlling for other variables included in the model, and does not reflect the correlation and causal relationship between variables. POMS has a negative impact on SES-2 (β = −0.013, *P* < 0.01), indicating that the influence of football player Mood on Sport Flow State is significant, but the impact is small, which means that the predictive ability of football player Mood on Sport Flow State is small. POMS also has a negative impact on CD-RISC (β = −0.734, *P* < 0.01), while CD-RISC has a positive impact on SES-2 (β = 0.019, *P* < 0.01), indicating that Mood in football players has a significant impact on Psychological Resilience, while Psychological Resilience in football players also has a significant mediating effect on Sport Flow State, but the actual impact is relatively small. POMS has a negative impact on AMS (β = −0.020, *P* < 0.01), while AMS has a positive impact on SES-2 (β = 0.432, *P* < 0.01). Mood of football players has a significant impact on Achievement Motivation in Competition, but the actual impact is relatively small, while Achievement Motivation in Competition of football players has a significant mediating effect on Sport Flow State, with an impact of 0.432; CD-RISC has a positive effect on AMS (β = 0.054, *P* < 0.01), indicating that Psychological Resilience of football players has a significant impact on Achievement Motivation in Competition, but the actual effect is relatively small. Based on the mutual influence between the above variables, it was jointly verified that Psychological Resilience and Achievement Motivation in Competition play a continuous mediating role in Mood and Sport Flow State of football players. This result validates hypothesis 2: Psychological resilience plays a mediating role in Mood and Sport Flow State of football players. The detailed path is shown in Figure 2.

**TABLE 2:** Regression analysis of variables,

Dependent variable	Variable	R	R^2^	F	β	T	*P*
CD-RISC	POMS	0.462	0.213	104.705	−0.734	−10.233	< 0.01
AMS	POMS	0.917	0.742	1023.599	−0.020	−8.207	< 0.01
	CD-RISC	–	–	–	0.054	35.673	< 0.01
SES-2	POMS	0.920	0.846	701.150	−0.013	−5.828	< 0.01
	CD-RISC	–	–	–	0.019	7.207	< 0.01
	AMS	–	–	–	0.432	10.263	< 0.01

The Bootstrap test results (Table 3) show that Psychological Resilience and Achievement Motivation in Competition partially mediate the relationship between the Mood and Sport Flow State of football players, with a total indirect effect of -0.040, accounting for 76.9% of the total effect. The mediating effect is composed of three indirect effects generated by pathways: (1) POMS → CD-RISC → SES-2 (r = −0.014, 95% CI -0.020∼-0.009), accounting for 26.9% of the total effect; (2) POMS → AMS → SES-2 (r = −0.009, 95% CI -0.013∼-0.005), accounting for 17.3% of the total effect; (3) POMS → CD-RISC → AMS → SES-2 (r = −0.017, 95% CI -0.022∼-0.013), accounting for 32.6% of the total effect.

**TABLE 3:** Total, direct, and indirect effects of intermediary testing.

Index	Effect	Boot SE	Boot LLCL	Boot ULCI
POMS → CD-RISC → SES-2	−0.014	0.003	−0.020	−0.009
POMS → AMS → SES-2	−0.009	0.002	−0.013	−0.005
POMS → CD-RISC → AMS → SES-2	−0.017	0.002	−0.022	−0.013
Total indirect effect	−0.040	0.003	−0.045	−0.034
Direct effect	−0.012	0.002	−0.017	−0.008
Total effect	−0.052	0.004	−0.056	−0.045

## 4 Discussion

### 4.1 The impact of Mood on Sports Flow State

The results of this study indicate that the Mood of football players has a significant negative impact on Sport Flow State (R = −0.013, *P* < 0.001), but the impact is very small. In the field of sports psychology research, it is generally believed that individual Mood have an impact on athletes’ participation experience and self goal positioning. This study also found that the Mood state of football players has a significant impact on their Sport Flow State during the game. The stronger the negative Mood of football players, the less smooth experience they obtain in sports. As a special psychological experience, the Sport Flow State is the subjective and objective perception interaction between individual athletes and the competition environment, social environment, or natural environment. When football players are confident, execute their skills and tactics smoothly, and receive sufficient team support during the game, they can form a smooth competition psychological experience and a sense of achievement satisfaction. This competitive psychological state is very important for the sports performance of football players (Yang, 2022). The pre competition psychological level of professional athletes is positively correlated with the frequency of smooth state during the competition, especially in high-pressure situations, where confident Mood can reduce hesitation in movements (Si et al., 2014). However, negative Moods may disrupt smooth states through attentional distraction (Cheng, 2022). The anxiety of adolescent athletes can also reduce visual search efficiency, leading to an increase in pass error rates (Xia, 2020). At the same time, players who maintain attention can more effectively concentrate their game energy, ignore irrelevant off field interference factors, thereby improving the efficiency and accuracy of information processing, which helps to enter the Sport Flow State (Liu et al., 2013). At present, cognitive intervention has been proven to be an effective psychological training method for optimizing the Mood state of football players and improving the frequency and quality of sports flow state generation. Through attention control training, self suggestion, thinking reconstruction and other methods, players can adjust their psychological state, optimize their cognitive structure, and better play their technical level in the game. Therefore, in the training and competition of football players, attention should be paid to the management and optimization of Mood status, in order to provide better psychological support and guarantee for athletes.

### 4.2 The mediating role of Psychological Resilience

The results of this study indicate that Psychological Resilience plays a mediating role in the Mood and Sport Flow State of football players, with a mediating effect of 26.9% of the total effect. Psychological Resilience is a personality trait in the field of sports psychology that describes the ability to maintain a high level of self-control, resist negative Mood, and ultimately achieve competition goals even in stressful situations. Football players with high Psychological Resilience can more effectively adjust their Mood state, maintain calmness and confidence, and thus better enter a smooth state in the face of low negative Mood. And this sports flow state manifests as a high level of focus, weakened self-awareness, disappearance of time awareness, and effective integration of movement and consciousness, which helps athletes perform at a high level in competitions (Gucciardi et al., 2021). Football players face enormous pressure in their daily training and matches, and the higher the level of competition, the greater the psychological pressure on the athletes. Individuals with high Psychological Resilience have stronger Mood regulation abilities and stronger adaptability and recovery abilities in the face of pressure or setbacks. Even in adversity, they can maintain good decision-making, confidence, and control over pressure and Mood, and steadily perform at their athletic level (Chang et al., 2012). These studies provide strong evidence for the mediating role of Psychological Resilience between Mood and Sport Flow State in football players. In the practice of football psychological training, it is important to focus on cultivating the psychological skills of football players and helping them establish positive cognitive models to cope with negative Mood during matches (Mansray, 2020). From this, it can be seen that improving athletes’ Psychological Resilience can effectively improve their Mood state, promote the generation of smooth state, and thus enhance their performance in competitions. Future research can further explore the specific influencing factors and training strategies of Psychological Resilience, providing more targeted guidance for the mental health and athletic performance of football players.

### 4.3 The mediating role of Achievement Motivation in Competition

This study found that the Achievement Motivation in Competition of football players plays a mediating role between Mood and Sport Flow State, with a mediating effect accounting for 17.3% of the total effect. In the field of competitive football, Achievement Motivation in Competition serves as an internal driving force for athletes to pursue excellence and surpass themselves, providing a new perspective for understanding the psychological dynamics of athletes during matches. Any type of sports motivation affects the direction of an athlete’s behavior, governs the intensity of their behavior, and their level of effort and persistence during training and competition, thereby affecting their athletic performance (Holden et al., 2017). At the same time, relevant scholars have verified the evidence of the relationship between adolescent motivation characteristics and elite performance levels of athletes, and found that players with highly intrinsic achievement oriented characteristics are more likely to achieve professional levels, which supports the importance of motivation characteristics in the mid to late stages of sports performance development (Zuber et al., 2022). And Achievement Motivation dominated by intrinsic motivation and mastery goals is usually positively correlated with stable and efficient athletic performance (Eikey, 2021). The mastery goal orientation of adolescent athletes can significantly predict the technical stability in competitions, as they focus more on skill improvement rather than short-term wins and losses (Halder and Phulkar, 2019). Intrinsic motivation helps athletes maintain their performance in high-pressure situations by enhancing their focus and resilience (Tanwer, 2015). The synergistic effect of Achievement Motivation and Psychological Resilience has also been shown to improve the speed of performance recovery in adversity (Carvalho et al., 2015). The evaluation of athletes’ Achievement Motivation has become an important psychological measurement basis for determining whether athletes can achieve professional or elite levels. In the psychological research of football players, measuring the Achievement Motivation of adolescent football players is very important. Relevant studies have confirmed the significant impact of Achievement Motivation on individual psychological stability and sports performance. Therefore, it is important to attach importance to the establishment and guidance of Achievement Motivation in Competition for football players, form a stable internal drive for sports achievement, and promote the sustainable development of football players.

### 4.4 The chain mediating role of Psychological Resilience and Achievement Motivation in Competition

This study found that Psychological Resilience and Achievement Motivation in Competition of football players play a chain mediated role between Mood and Sport Flow State, with the chain mediated effect accounting for 32.6% of the total effect. Psychological Resilience and Achievement Motivation in Competition are important psychological factors for football players to participate in competitions. Football players with higher levels of Psychological Resilience and Achievement Motivation in Competition are better able to resist the negative impact of negative Mood on the Sport Flow State. Related studies have shown that Psychological Resilience indirectly enhances athletes’ Achievement Motivation by improving Moodal regulation efficiency (Sarkar and Fletcher, 2014). And a study on teenage football players shows that, Psychological Resilience. The chain pathway of Moodal stability and intrinsic motivation can explain changes in motor performance (Mistretta et al., 2017). Psychological Resilience first promotes challenging goal setting, thereby strengthening the persistence of Achievement Motivation. Athletes with high Psychological Resilience are more inclined to adjust their strategies rather than give up when their goals are obstructed, and this goal resilience partially mediates the relationship between Psychological Resilience and Achievement Motivation (Mathéo and Martinent, 2023). At the same time, research has found that reducing athletes’ competitive anxiety during competitions is beneficial. The impact of athletes’ pre competition Mood on competition psychology is mainly related to coping with adversity and psychological control under pressure. Higher self-esteem of athletes is a prerequisite for better preparation for competitions, which is related to appropriate goal setting, guidance, and Achievement Motivation. Fear of failure and loss of athlete reputation can disrupt athletes’ attention during competitions (Adriana, 2024). From this, it can be seen that Psychological Resilience is closely related to Achievement Motivation in Competition, and the interaction between the two can affect the impact of football players’ Mood on the Sport Flow State.

## 5 Conclusion

Football player Mood has a significant negative impact on Sport Flow State, but its actual predictive ability for Sport Flow State is very small. Psychological Resilience also has a significant mediating effect on the Sport Flow State, but the actual impact is relatively small. Achievement Motivation in Competition has a significant mediating effect on the Sport Flow State, but the actual impact is general. Psychological Resilience and Achievement Motivation in Competition play a chain mediated role between the Mood and Sport Flow State of football players.

### 5.1 Research limitations

This study has certain limitations: firstly, it only explored the mediating mechanism between Psychological Resilience and Achievement Motivation in Competition, without addressing individual differences. Future research can further explore the relationship between football players’ Mood and Sport Flow State from the perspective of regulatory mechanisms; Secondly, this study only used college students from Sichuan Province, China, and the representativeness and richness of the sample need to be improved. Future research can investigate other different regions and age groups of participants to further test the applicability of the results; Finally, due to limited time and funding, this study is a horizontal research and it is difficult to determine the exact causal relationship. In the future, a more in-depth analysis of the relationship between the two can be conducted through a longitudinal research design.

## Data Availability

The original contributions presented in this study are included in this article/Supplementary material, further inquiries can be directed to the corresponding author.
