# Supplementary material for: The impact of Mood on Sports Flow State in football players: a chain mediating model of Psychological Resilience and Achievement Motivation in Competition
Source: Front Psychol. 2025 May 9;16:1523400. doi: 10.3389/fpsyg.2025.1523400 (PMC12098437; doi:10.3389/fpsyg.2025.1523400)
Supplement: Supplementary file 2 [file Presentation_1.PDF]

```
GET
  FILE='D:\deskep\十一月份投稿2\data.sav'.
DATASET NAME 数据集1 WINDOW=FRONT.
DESCRIPTIVES VARIABLES=POMS SES2 CDRISC AMS
  /STATISTICS=MEAN STDDEV.
```

**描述统计**

| | 个案数 | 平均值 | 标准差 |
|---|---|---|---|
| POMS | 388 | 94.374 | 7.8053 |
| SES-2 | 388 | 3.7029 | .68976 |
| CD-RISC | 388 | 52.680 | 12.4005 |
| AMS | 388 | 3.5723 | .82600 |
| 有效个案数（成列） | 388 | | |

```
CORRELATIONS
  /VARIABLES=POMS SES2 CDRISC AMS
  /PRINT=TWOTAIL NOSIG
  /MISSING=PAIRWISE.
```

**相关性**

| | | POMS | SES-2 | CD-RISC | AMS |
|---|---|---|---|---|---|
| POMS | 皮尔逊相关性 | 1 | -.591** | -.462** | -.564** |
| | 显著性（双尾） | | .000 | .000 | .000 |
| | 个案数 | 388 | 388 | 388 | 388 |
| SES-2 | 皮尔逊相关性 | -.591** | 1 | .871** | .903** |
| | 显著性（双尾） | .000 | | .000 | .000 |
| | 个案数 | 388 | 388 | 388 | 388 |
| CD-RISC | 皮尔逊相关性 | -.462** | .871** | 1 | .902** |
| | 显著性（双尾） | .000 | .000 | | .000 |
| | 个案数 | 388 | 388 | 388 | 388 |
| AMS | 皮尔逊相关性 | -.564** | .903** | .902** | 1 |
| | 显著性（双尾） | .000 | .000 | .000 | |
| | 个案数 | 388 | 388 | 388 | 388 |

**. 在 0.01 级别（双尾），相关性显著。

```
/* PROCESS version 3.3 */.
/* Written by Andrew F. Hayes */.
/* www.afhayes.com */.
/* www.processmacro.org */.
/* Copyright 2019 by Andrew F. Hayes */.
/* Documented in http://www.guilford.com/p/hayes3 */.
/* PROCESS workshop schedule at http://www.processmacro.org/workshops.html */.

/* Distribution of this code in any form, except through processmacro.org, is prohibited */.
/* without the permission of the copyright holder */.

/* THIS SOFTWARE IS PROVIDED "AS IS", WITHOUT WARRANTY OF ANY KIND */.
/* EXPRESS OR IMPLIED, INCLUDING BUT NOT LIMITED TO THE WARRANTIES OF */.
/* MERCHANTABILITY, FITNESS FOR A PARTICULAR PURPOSE AND NONINFRINGEMENT */.
/* IN NO EVENT SHALL THE COPYRIGHT HOLDERS BE LIABLE FOR ANY CLAIM, */.
/* DAMAGES OR OTHER LIABILITY, WHETHER IN AN ACTION OF CONTRACT, TORT */.
/* OR OTHERWISE, ARISING FROM, OUT OF OR IN CONNECTION WITH THE */.
/* SOFTWARE OR THE USE OR OTHER DEALINGS IN THE SOFTWARE */.
/* USE OF THIS SOFTWARE IMPLIES AGREEMENT WITH THESE TERMS */.

set printback=off.
```

Run MATRIX procedure:

```
*************** PROCESS Procedure for SPSS Version 3.3 ******************

       Written by Andrew F. Hayes, Ph.D.     www.afhayes.com
   Documentation available in Hayes (2018). www.guilford.com/p/hayes3

**************************************************************************
Model  : 6
   Y  : SES2
   X  : POMS
  M1  : CDRISC
  M2  : AMS
```

Sample
Size: 388

**************************************************************************
OUTCOME VARIABLE:
CDRISC

Model Summary

| R | R-sq | MSE | F | df1 | df2 | p |
|---|---|---|---|---|---|---|
| .462 | .213 | 121.275 | 104.705 | 1.000 | 386.000 | .000 |

Model

| | coeff | se | t | p | LLCI | ULCI |
|---|---|---|---|---|---|---|
| constant | 121.940 | 6.792 | 17.955 | .000 | 108.587 | 135.293 |
| POMS | -.734 | .072 | -10.233 | .000 | -.875 | -.593 |

Standardized coefficients

| | coeff |
|---|---|
| POMS | -.462 |

**************************************************************************
OUTCOME VARIABLE:
AMS

Model Summary

| R | R-sq | MSE | F | df1 | df2 | p |
|---|---|---|---|---|---|---|
| .917 | .842 | .109 | 1023.599 | 2.000 | 385.000 | .000 |

Model

| | coeff | se | t | p | LLCI | ULCI |
|---|---|---|---|---|---|---|
| constant | 2.584 | .275 | 9.389 | .000 | 2.043 | 3.126 |
| POMS | -.020 | .002 | -8.207 | .000 | -.025 | -.015 |
| CDRISC | .054 | .002 | 35.673 | .000 | .051 | .057 |

Standardized coefficients

| | coeff |
|---|---|
| POMS | -.188 |
| CDRISC | .816 |

**************************************************************************
OUTCOME VARIABLE:

SES2

Model Summary

| R | R-sq | MSE | F | df1 | df2 | p |
|---|---|---|---|---|---|---|
| .920 | .846 | .074 | 701.150 | 3.000 | 384.000 | .000 |

Model

| | coeff | se | t | p | LLCI | ULCI |
|---|---|---|---|---|---|---|
| constant | 2.361 | .252 | 9.368 | .000 | 1.865 | 2.856 |
| POMS | -.013 | .002 | -5.828 | .000 | -.017 | -.008 |
| CDRISC | .019 | .003 | 7.207 | .000 | .014 | .024 |
| AMS | .432 | .042 | 10.263 | .000 | .349 | .515 |

Standardized coefficients

| | coeff |
|---|---|
| POMS | -.143 |
| CDRISC | .338 |
| AMS | .517 |

************************** TOTAL EFFECT MODEL ****************************

OUTCOME VARIABLE:

SES2

Model Summary

| R | R-sq | MSE | F | df1 | df2 | p |
|---|---|---|---|---|---|---|
| .591 | .349 | .310 | 207.028 | 1.000 | 386.000 | .000 |

Model

| | coeff | se | t | p | LLCI | ULCI |
|---|---|---|---|---|---|---|
| constant | 8.631 | .344 | 25.115 | .000 | 7.955 | 9.306 |
| POMS | -.052 | .004 | -14.388 | .000 | -.059 | -.045 |

Standardized coefficients

| | coeff |
|---|---|
| POMS | -.591 |

************** TOTAL, DIRECT, AND INDIRECT EFFECTS OF X ON Y **************

Total effect of X on Y

| Effect | se | t | p | LLCI | ULCI | c_ps | c_cs |
|---|---|---|---|---|---|---|---|
| -.052 | .004 | -14.388 | .000 | -.059 | -.045 | -.076 | -.591 |

Direct effect of X on Y

| Effect | se | t | p | LLCI | ULCI | c'_ps | c'_cs |
|---|---|---|---|---|---|---|---|
| -.013 | .002 | -5.828 | .000 | -.017 | -.008 | -.018 | -.143 |

Indirect effect(s) of X on Y:

| | Effect | BootSE | BootLLCI | BootULCI |
|---|---|---|---|---|
| TOTAL | -.040 | .003 | -.046 | -.034 |
| Ind1 | -.014 | .003 | -.020 | -.009 |
| Ind2 | -.009 | .002 | -.014 | -.005 |
| Ind3 | -.017 | .002 | -.022 | -.013 |
| (C1) | -.005 | .005 | -.015 | .004 |
| (C2) | .003 | .004 | -.006 | .011 |
| (C3) | .009 | .002 | .004 | .013 |

Partially standardized indirect effect(s) of X on Y:

| | Effect | BootSE | BootLLCI | BootULCI |
|---|---|---|---|---|
| TOTAL | -.057 | .004 | -.065 | -.051 |
| Ind1 | -.020 | .004 | -.029 | -.013 |
| Ind2 | -.012 | .003 | -.020 | -.007 |
| Ind3 | -.025 | .003 | -.031 | -.019 |
| (C1) | -.008 | .007 | -.022 | .006 |
| (C2) | .005 | .006 | -.009 | .016 |
| (C3) | .013 | .003 | .005 | .019 |

Completely standardized indirect effect(s) of X on Y:

| | Effect | BootSE | BootLLCI | BootULCI |
|---|---|---|---|---|
| TOTAL | -.448 | .025 | -.498 | -.401 |
| Ind1 | -.156 | .033 | -.227 | -.098 |
| Ind2 | -.097 | .025 | -.152 | -.052 |
| Ind3 | -.195 | .023 | -.241 | -.150 |
| (C1) | -.059 | .055 | -.169 | .046 |
| (C2) | .039 | .050 | -.066 | .129 |
| (C3) | .098 | .026 | .042 | .146 |

Specific indirect effect contrast definition(s):

| | | | |
|---|---|---|---|
| (C1) | Ind1 | minus | Ind2 |
| (C2) | Ind1 | minus | Ind3 |
| (C3) | Ind2 | minus | Ind3 |

Indirect effect key:

```
Ind1 POMS      ->   CDRISC    ->   SES2
Ind2 POMS      ->   AMS       ->   SES2
Ind3 POMS      ->   CDRISC    ->   AMS       ->   SES2

*********************** ANALYSIS NOTES AND ERRORS ************************

Level of confidence for all confidence intervals in output:
  95.0000

Number of bootstrap samples for percentile bootstrap confidence intervals:
  5000

------ END MATRIX -----
```
